# Pandemic influenza: implications for occupational medicine

**DOI:** 10.1186/1745-6673-4-15

**Published:** 2009-06-23

**Authors:** W Shane Journeay, Matthew D Burnstein

**Affiliations:** 1 Dalhousie Medical School, Dalhousie University, Faculty of Medicine, Halifax, Nova Scotia, B3H 4H7 Canada; 2 Bell-Aliant Health & Wellness Division, 1505 Barrington Street, Halifax, Nova Scotia, B3J 3K5 Canada

## Abstract

This article reviews the biological and occupational medicine literature related to H5N1 pandemic influenza and its impact on infection control, cost and business continuity in settings outside the health care community. The literature on H5N1 biology is reviewed including the treatment and infection control mechanisms as they pertain to occupational medicine. Planning activity for the potential arrival of pandemic avian influenza is growing rapidly. Much has been published on the molecular biology of H5N1 but there remains a paucity of literature on the occupational medicine impacts to organizations. This review summarizes some of the basic science surrounding H5N1 influenza and raises some key concerns in pandemic planning for the occupational medicine professional. Workplaces other than health care settings will be impacted greatly by an H5N1 pandemic and the occupational physician will play an essential role in corporate preparation, response, and business continuity strategies.

## Introduction

The occupational medicine community has been adressing occupational diseases of epidemic proportions since Ramazzini first studied injured workers. Traditionally, these diseases have been musculoskeletal, psychiatric or toxicologic in nature. When the etiology of these conditions has been identified, appropriate measures have been taken to mitigate the risk of becoming ill or injured. Occupational health specialists are therefore quite adept at looking at prevention when the causative factors are known and their mechanism of action understood. However, when the process is poorly understood, as is the case with pandemic influenza, determining the most appropriate prevention and mitigation strategy is more complex. Despite this uncertainty, government agencies and businesses are taking measures to address the impact of a potential pandemic influenza on their workforce [1,2]. The field of occupational medicine is being consulted to assist in mitigating the impact of an avian influenza pandemic on their human resources, business continuity and also the societal impact associated with essential services and disease transmission. This article will outline the nature of pandemic avian influenza and some of the unique considerations related to the occupational environment outside the health care setting.

### Learning from SARS

Occupational medicine professionals are uniquely positioned to provide information on the potential impact of a pandemic influenza. Indeed, infectious disease may disproportionately impact the occupational environment. This is due to factors associated with transmission such as the proximity of co-workers to one another in the workplace, during the daily commute to work, or simply dealing face to face with customers. Of particular concern is the health and safety of those health care professionals caring for infected patients. The recent experience with Severe Acute Respiratory Syndrome (SARS) provides some useful insight into the consequences of a novel infection on a modern society and more specifically on the health care community.

There are many similarities between the SARS epidemic and the anticipated experience with avian influenza. Both have been associated with food and animals. In the early stages of SARS, more than a third of infected humans were food handlers [3], and it was later inferred that the SARS coronavirus had originated in civet cats, and that the first transmission of infection to humans may have occurred in those workers handling civet cats [4]. However, the greatest impact of SARS was subsequently felt in health care workers where they were estimated to have accounted for over 20% of total SARS cases in Singapore and 40% in Canada [5]. Thus, not only are individuals working closely with infected animal hosts at risk for first line crossover transmission of an emerging virus but they are also at risk of acquiring the virus from coworkers, or in the case of health care professionals, from patients.

### Influenza Virology

Influenza are single stranded RNA viruses and are part of the Orthomyxoviradae family [6]. Influenza A and B can recur in individuals because of their ongoing mutation. Antigenic drifts can occur in seasonal influenza and if sufficient mutations arise in the surface proteins Hemagluttinin or Neuraminidase, it can result in a novel strain. Thus 'H' and 'N' components determine the different potential subtypes of a given influenza virus, and at present a total of 15 H variants exist while 9 N subtypes have been identified. The ongoing emergence of small but significant mutations can lead to epidemics which we experience as seasonal influenza. The yearly influenza vaccination program is based on correctly determining which of these subtle changes (drift) will become predominant. Pandemic influenza, such as the suspected H5N1avian influenza, occurs as a result of major changes in surface proteins of influenza viruses known as an antigenic shift. This novel strain which is present in animals still requires further modification before it can effectively spread among the human population. This situation can be created via transmission from a different species with frequent exposure leading to adaptation, or from genetic reassortment [6]. The process of reassortment happens when an individual simultaneously has both human and avian influenza subtypes. This allows for a recombination of viral components, leading to a new viral form with the potential for efficient transmission between humans. This form of the virus would still contain avian viral surface proteins. When this occurs humans have minimal or no immunity against the virus, enabling a large geographic spread of disease with high attack rates [6,7]. It should be noted that H5N1 is not the only avian influenza that has the capacity to affect humans. H7N2 is slowly progressing globally, and while less pathogenic than H5N1, has caused illness in poultry workers. To date, neither of these avian influenzas has gained the capacity to spread efficiently from human to human.

### Pandemic influenza

Pandemic influenza occurs when a new strain of human influenza arises that humans have minimal or absent pre-existing natural immunity, which causes disease, can be easily transmitted from person to person, and is globally widespread (on 3 continents at one time) [7] or exhibits community level outbreaks in two WHO regions. In today's globalized economy and interdependent supply chain, the work force is particularly sensitive to pandemic infections and it is also a key mechanism for the geographic spread of a pandemic. On average, we experience a pandemic about every thirty years. Indeed, in the 20^th ^century, there were three pandemic influenza outbreaks which included: the Spanish Influenza (1918–1920), Asian Influenza (1957–1958) and the Hong Kong Influenza (1968–1969) [8]. This is not to suggest that simply because 30 years have passed since the last pandemic, we are overdue; it is simply meant to point out that pandemics are relatively common events given the right conditions. The current strain of influenza considered to have pandemic potential is the highly pathogenic H5N1 strain of avian influenza which has spread from Asia to Europe. Moreover, its transmission to humans has intensified concerns that a novel strain will emerge leading to human infections of pandemic proportions [7]. The three criteria that are required to enable a pandemic include: 1) the presence of a new viral strain that is capable of infecting humans, 2) ability to be transmitted from person to person, and 3) availability of a susceptible global population [6]. Thus, should a new viral strain emerge, the global workforce provides and ideal vehicle in which transmission from person to person can occur within a susceptible global population. The ability of H5N1 to propagate between humans after an initial infection has not been established and its probability is unknown. Thus avian influenza has currently not developed into a pandemic [6,9]. However, it is generally accepted that this will occur; it is a matter of "when, not if". When this occurs, the health care system will be particularly susceptible to pandemic influenza events. This is because patients with influenza will place an enormous burden on already fully taxed health care services and because health care professionals will come into direct contact with infected patients rendering them susceptible to acquiring the virus. However, there are no industries that would be left unaffected by an avian influenza pandemic, and therefore public health agencies, government, and industry will need to consider the level of interdependence they share.

### Transmission

It is generally accepted that transmission of the influenza virus occurs by host inhalation of viral droplets usually greater than 5 μm in size [7,10]. A recent review of the mechanism of influenza transmission concluded that the virus is primarily transmitted at close quarters [11]. It can also be transmitted by coming into contact with viral laden fomites. Both of these methods are of great concern in the workplace, due to use of communal equipment and also in areas where employees work in close proximity. Therefore, infection control measures will need to vary between industries. For example, staff that work in isolation or even outdoors could be at far less risk of transmission than having many employees in a single room such as a telecommunications call center where individuals are separated by small distances. Moreover, unlike seasonal influenza which has an incubation period of one to four days (average two), avian influenza has an incubation period ranging from two to eight days [12]. This has implications for staffing schedules and return to work policy when developing guidelines for pandemic influenza in the workplace. Once again the nature of the control measures and advisement to employees may vary considerably depending on the physical layout of the worksite.

### The workplace as a transmission center

It is well established that occupational disease is already an enormous contributor to the economic and human resource strain on our health care systems. Many mechanisms are in place to prevent or manage such disease which may include ergonomic initiatives, exposure limits, and corporate health and wellness programs. At the same time, the workplace is one of the key pillars of societal function, such that the health of a workplace is vital to the health and functioning of our interdependent society. This is particularly true when one considers such essential services as health care, energy, communications, and food supply sectors.

In the event of a pandemic influenza absenteeism will be an enormous challenge. Employees will not be present due to reasons such as: infection and illness from the pandemic influenza strain, exclusion from work while suffering an illness that is mistaken for or treated empirically as influenza, caring for sick relatives, caring for children in the event of day care and school closures by governments, loss of public transportation and based on the fear of real or perceived risk of infection at work or during travel [13]. The Public Health Agency of Canada is predicting total work absenteeism of 35 to 50% during the whole disease wave with the peak work absence ranging from 15 to 27%.

While it is tempting to look at absenteeism from within a single organization, the functioning of a company is almost always dependent on external clients, supply chains, or multi-national locations. Thus, a large manufacturing plant in United States may require final product detailing in another region of the country, which in turn receives its raw materials from Asia or South America. "Just on time" delivery processes have created a society in which most companies (including health care institutions) have less than a few weeks supply of essential goods (including medications). Little is known about the global timing and progression of H5N1 avian influenza at present but it is entirely possible that while an organization in North America is healthy, its supplier abroad is experiencing a disease wave leading to uncoordinated business efforts. Each company has an obligation to ensure that occupational transmission is attenuated and planned for, but this will also require cooperation with governments that may impose social and travel restrictions to suppress the spread of the disease while still maintaining business continuity and societal function. Pandemic influenza, will have the capacity to disrupt services and supply chains and thus requires significant planning and foresight from occupational medicine professionals to help mitigate the health and economic impacts to their organizations and to the functioning of society [14].

### Infection controls

As with any occupational disease, the interventions available to health professionals can be considered as engineering or administrative controls. As well, pharmaceutical controls (prophylaxis) for avian influenza may provide an important role in prevention. However, there is limited clinical evidence for the effectiveness of currently available medications or vaccines.

#### Vaccines

Vaccination strategies, such as the annual influenza vaccine programs, have been the traditional first line of defense against viral infections. Research is currently being devoted to the development of vaccines as a possible intervention for pandemic influenza. The need for a rapidly deliverable vaccine for pandemic influenza has become more urgent since de Jong et al. [15] reported the emergence of oseltamivir resistance to H5N1. Given the current 4 to 6 month development time, it is unlikely that a vaccination will be available during the first wave of a pandemic. The impact of antigenic drift on vaccination for influenza is an on ongoing challenge and is the reason vaccination for seasonal influenza must be administered annually to protect against the new antigenic strain. Increased demand for vaccine during a pandemic influenza may be tempered by the supply. Specifically, the substrate used for vaccine manufacturing for all major suppliers worldwide is chicken eggs [16]. During a pandemic several times the current supply of eggs would be required. What is even more challenging is that H5N1, which is the current predicted pandemic strain, is lethal in eggs and is also a biosafety level 3 pathogen which decreases the potential of scaling up the manufacture of vaccine for international deployment [16]. One must also consider that poultry workers may be at increased risk of exposure to pandemic influenza zoonotically or may also be stretched from a human resource perspective when measures need to be taken to curb a poultry influenza outbreak [17]. Acambis Labs, and others, are working on the development of a universal influenza vaccination that is based on more stable surface proteins such as M2e, which is found on the surface of all influenza A strains.

The first vaccine approved by the US food and Drug Administration for pandemic influenza is a reverse genetics vaccine and demonstrated low immunogenicity except for high doses with an adjuvant [18]. When this was approved by the FDA it was noted that the vaccine would not be marketed to the general public but rather stockpiled by governments[16]. It has previously been suggested that an appropriate vaccine will likely not be determined until the initial phase of a pandemic [19]. Furthermore, once a vaccine is developed a mechanism needs to be put in place that can provide an adequate supply at an affordable cost globally in lock step with the progression of the pandemic.

A unique challenge for the occupational medicine physician in the event of a pandemic outbreak is to determine who gets priority for receiving vaccination. Maintenance of essential services will be central to the continuity of a functioning society. Health care workers and workers in critical occupations will be a priority for vaccination programs, once available. Decisions on vaccination programs are complicated by the eventual timing of the disease wave, number of employees, nature of the work environment, and the availability of vaccine. For example, should employees who are in close proximity to one another be given priority or only those critical to maintaining business continuity? The Public Health Agency of Canada has created priority lists for receipt of vaccinations [20]. Not surprisingly health care workers are part of group 1, followed by key societal decision makers and critical protection and utility workers (police, fire fighters, sewage workers, public transportation and communications).

#### Supplying anti-virals

Another treatment option is the use of anti-viral medications. The two main classes of antivirals available at present are the neuraminidase inhibitors and the adamantanes. There has been an emergence of resistance to adamantanes for seasonal influenza [21] leading many to reconsider them as agents in the treatment of pandemic avian influenza [22]. In preliminary studies using oseltamivir [23] or zanamivir [24], patients showed a reduction in the duration of symptoms ranging from 1–2 days. Whether a 1–2 day reduction in symptoms will translate into reduced absenteeism, cost-savings and disease transmission is unknown. Additionally, the cost-benefit of stockpiling anti-virals for treatment of pandemic influenza remains unknown. As noted previously, oseltamivir has also demonstrated resistance [15]. Adding to the complexity of managing H5N1 treatment, is once again the manner in which one decides who receives the medication and the fact that the modest reduction in influenza symptoms will depend on timing of administration of the drug. In individuals with confirmed H5N1 influenza that were treated with oseltamivir, mortality was still close to 80% [25]. It has also been noted by Tambyah [22], that despite guidelines from the World Health Organization concerning the use of anti-virals in pandemic avian influenza, there remains little 'level 1' clinical evidence to support such guidelines. More recently, a group in Singapore has gathered a set of practical guidelines for clinicians encountering H5N1 avian influenza in humans [26]. Despite the lack of scientific evidence for their effectiveness in a pandemic situation, governments and many employers are stockpiling anti-virals to be used not only as therapy for ill individuals, but also as prophylaxis for critical staff. This may be driven by the recognition that once the pandemic is recognized, it will be nearly impossible to purchase these products. It reflects a significant investment: at approximately $3/pill, an eight week course would cost over $200 per employee. A company of 1000 employees would need to invest $200,000 on a product which they hope they will never use, is unproven, and has a limited shelf life. Again, one is faced with decisions regarding dispensing medication – to all workers, critical workers, families?

#### Non-pharmaceutical controls

While the world waits for an effective pharmaceutical intervention, non-pharmaceutical controls will need to be considered to combat the spread of illness in the community and the workplace.

Low [7] has outlined and adapted [27] five non-pharmaceutical public health interventions that would aid in the mitigation of pandemic influenza. They include: hand hygiene and respiratory etiquette, human surveillance, rapid viral diagnosis, provider and patient use of masks and other personal protective equipment and isolation of the sick. All of these interventions will need to be coordinated at organizational and government levels due to the tremendous interrelationships affected by a pandemic. Some of the above interventions have some unique implications from an occupational medicine perspective.

Hygiene and respiratory etiquette are particularly effective in reducing the spread of infectious disease and represent a key defense against nosocomial infection in hospitals. This also applies to a workplace where people are in close proximity to one another where viral droplets may exist in the air and on equipment or surfaces used by multiple people each day. The spread of infection between employees is one possible transmission pathway, however the occupational medicine professionals of large and complex organizations must also consider the families of the employees and the consumers of products where interaction occurs with the public. Protection of the consumer raises the issue of due diligence which can be complex for service oriented organizations. Hand washing, social distancing and respiratory etiquette, if normalized and rigorously adopted, may provide the most effective (certainly most cost effective) means of protection.

### N95 Respirators

The role of personal protective equipment in reducing the spread of pandemic influenza is one of considerable debate. Both the perceived and/or real efficacy of such measures and the cost associated with the provision of such materials are legitimate concerns for those coordinating pandemic plans in the workplace. The gold standard for particulate inhalation in most cases is the use of the N95 respirator. Droplet transmission is thought to be the primary mode of transmission and is the basis of guidelines for health professionals coming within 3 feet of patients during seasonal influenza [7,28]. Therefore, because N95 respirators can trap more than 95% of airborne particles [28,29], experience from their use in seasonal influenza supports some effectiveness of their application to pandemic avian influenza.

Regardless of the real or perceived protection that N95 respirators provide to employees from transmitting or contracting H5N1 influenza via inhalation, many challenges exist with the use of such protective equipment. N95 respirators require fit testing, need to be replaced, and tend to be uncomfortable which create opportunities for their improper and therefore ineffective use. Moreover, the N95 respirators would impose a large cost to an organization who decides they will outfit their employees with them in the event of a pandemic. This cost is imposed by buying a stockpile of the respirators, and the provision of fit-testing for each and every employee issued a respirator. Consider an organization that decides that during a two week pandemic disease wave they will issue N95 masks to 1000 employees. Each respirator unit has a cost of $1, and because the respirators need to be changed every 2–3 hours, each employee working an 8-hour day will require 3 masks per day. Therefore, each employee would require 30 masks over 2-weeks (10 working days), leading to a cost of $30 per employee for a total of $30K for 1000 employees for two weeks. This does not include the cost associated with fit-testing which takes approximately 20-minutes per person, which would therefore require 333 hours of time to fit test 1000 employees. Furthermore, a trained professional is required to perform the fit testing procedure. Finally, does the employer provide N95 masks for the families of the employees such that protection is afforded to the family and the employee at home? All of these measures will vary as the risk of transmission will depend upon the nature of the worksite and the controls put in place. For example, teleworking would greatly reduce the number of employees that congregate at the worksite. Not all industries will have this luxury.

Creating an environment in which employees are comfortable and confident of their safety in the workplace is critical in enhancing their work attendance. Fear will be rampant, and employee education well in advance of the event will be vital in reducing the spread of disease, myths, and ensuring corporate and social stability. Indeed addressing both real and perceived risk of infection may be the most crucial factor in maintaining business continuity in the face of a pandemic.

## Conclusion

The scientific community is devoting a great deal of effort and research funding towards what is considered by many to be an inevitable pandemic. It has also been suggested that even the most stringent non-pharmaceutical interventions are unlikely to prevent the pandemic or alter the underlying biological susceptibility of a population to a pandemic virus [7]. However, the prevention and management of disease transmission in the occupational environment will play a central role in the health and economic burden of pandemic influenza. With a long-standing record of applying the latest science to appropriate engineering and administrative disease controls, the occupational medicine community can utilize these concepts to prepare for and mitigate the potential impact on industry and society.

## Appendix

At the time this paper was submitted to this journal the WHO and many governments are monitoring an outbreak of H1N1 swine influenza which has recently been declared a pandemic. Cases have been confirmed here in Nova Scotia, United States, UK, Spain and Israel with the epicenter in Mexico where over 100 people have died. While much of the literature focused on the future possibility of H5N1 avian influenza pandemic, the H1N1 swine influenza strain was not of immediate concern to the international community until the current outbreak in Mexico.

## Competing interests

The authors declare that they have no competing interests.

## Authors' contributions

WSJ conceived, researched, wrote and edited the manuscript. MDB provided background information, guidance and editing. Both authors reviewed and approved the final submitted manuscript.

## References

[B1] Smith PW, Hansen K, Spanbauer L, Sheil DF (2007). Pandemic influenza preparedness: A survey of businesses. Am J Infect Control.

[B2] Bartlett JG (2006). Planning for avian influenza. Ann Intern Med.

[B3] WHO/CDS/CSR/GAR/2003.11 (2003). Consensus document on the epidemiology of severe acute respiratory syndrome (SARS). http://www.who.int/csr/sars/en/WHOconsensus.pdf.

[B4] Kan B, Wang M, Jing H, Xu H, Jiang X, Yan M, Liang W, Zheng H, Wan K, Liu Q, Cui B, Xu Y, Zhang E, Wang H, Ye J, Li G, Li M, Cui Z, Qi X, Chen K, Du L, Gao K, Zhao YT, Zou XZ, Feng YJ, Gao YF, Hai R, Yu D, Guan Y, Xu J (2005). Molecular evolution analysis and geographic investigation of severe acute respiratory syndrome coronavirus-like virus in palm civets at an animal market and on farms. J Virol.

[B5] Koh D (2005). Emerging infections among health care workers: the severe acute respiratory syndrome (SARS) experience. GOHNET Newsletter.

[B6] Lee VJ, Fernandez GG, Chen MI, Lye D, Leo YS (2006). Influenza and the pandemic threat. Singapore Med J.

[B7] Low DE (2008). Pandemic planning: Non-pharmaceutical interventions. Respirology.

[B8] Johnson NP, Mueller J (2002). Updating the accounts: global mortality of the 1918–1920 'Spanish' influenza pandemic. Bull Hist Med.

[B9] Halpin J (2005). Avian flu from an occupational health perspective. Arch Environ Occup Health.

[B10] Cate TR (1987). Clinical manifestations and consequences of influenza. Am J Med.

[B11] Brankston G, Gitterman L, Hirji Z, Lemieux C, Gardam M (2007). Transmission of influenza A in human beings. Lancet Infect Dis.

[B12] Beigel JH, Farrar J, Han AM, Hayden FG, Hyer R, de Jong MD, Lochindarat S, Nguyen TK, Nguyen TH, Tran TH, Nicoll A, Touch S, Yuen KY (2005). Avian influenza A (H5N1) infection in humans. The Writing Committee of the World Health Organization (WHO) Consultation on Human Influenza A/H5. New Engl J Med.

[B13] Dalton CB (2006). Business continuity management and pandemic influenza. N S W Public Health Bull.

[B14] Maldin-Morgenthau B, Toner C, Wilkinson D, Horwitz K, Atons K, Inglesby TV, O'Toole T (2007). Roundtable discussion: Corporate Pandemic Preparedness. Biosecur Bioterror.

[B15] de Jong MD, Tran TT, Truong HK, Vo MH, Smith GJ, Nguyen VC, Bach VC, Phan TQ, Do QH, Guan Y, Peiris JS, Tran TH, Farrar J (2005). Oseltamivir resistance during treatment of influenza A (H5N1) infection. New Engl J Med.

[B16] Tambyah PA (2008). Update on influenza vaccines. Respirology.

[B17] Gray GC, Trampel DW, Roth JA (2007). Pandemic influenza planning: Shouldn't swine and poultry workers be included?. Vaccine.

[B18] Bresson JL, Peronne C, Launay O, Gerdil c, Saville M, Wood J, Höschler K, Zambon MC (2006). Safety and immunogenicity of an inactivated split-viron influenza A/Vietnam/1194/2004 (H5N1) vaccine: phase I randomised trial. Lancet.

[B19] Webby RJ, Webster RG (2003). Are we ready for pandemic influenza?. Science.

[B20] Public Health Agency of Canada (2008). Preparing for the pandemic vaccine response – Annex D.

[B21] Barr IG, Hurt AC, Deed N, Iannello P, Tomasov C, Komadina N (2007). The emergence of adamantane resistance in influenza A (H1) viruses in Australia and regionally in 2006. Antiviral Res.

[B22] Tambyah PA (2008). Update on influenza anti-virals. Respirology.

[B23] Nicholson KG, Aoki FY, Osterhaus AD, Trottier S, Carewicz O, Mercier CH, Rode A, Kinnersley N, Ward P (2000). Efficacy and safety of oseltamivir in treatment of acute influenza: a randomised controlled trial. Neuraminidase Inhibitor Flu Treatment Investigation Group. Lancet.

[B24] Lalezari J, Compion K, Keene O, Silagy C (2001). Zanamivir for treatment of influenza A and B infection in high-risk patients: a pooled analysis of randomized controlled trials. Arch Intern Med.

[B25] (WHO). The Working Committee of the WHO (2005). Consultation on human influenza A/H5 avian influenza A (H5N1) infection in humans. New Engl J Med.

[B26] Lye DC, Nguyen DH, Giriputro S, Anekthananon T, Eraksoy H, Tambyah PA (2006). Practical management of avian influenza in humans. Singapore Med J.

[B27] Aledort JE, Lurie N, Wasserman J, Bozzette SA (2007). Non-pharmaceutical public health interventions for pandemic influenza: an evaluation of the evidence base. BMC Public Health.

[B28] Bridges CB, Kuehnert MJ, Hall CB (2003). Transmission of influenza: implications for control in health care settings. Clin Infect Dis.

[B29] Low DE, Bartlett K, Baudouin J-L, Bourgault AM, Brosseau L (2007). Influenza Transmission and the Role of Personal Protective Respiratory Equipment: An Assessment of Evidence.

